# Functional magnetic resonance imaging (fMRI) as adjunct for planning laser interstitial thermal therapy (LITT) near eloquent structures

**DOI:** 10.1007/s00701-024-05970-x

**Published:** 2024-02-05

**Authors:** Ziad Rifi, Maya Harary, Patricia D. Walshaw, Andrew J. Frew, Richard G. Everson, Aria Fallah, Noriko Salamon, Won Kim

**Affiliations:** 1 David Geffen School of Medicine, University of California, Los Angeles, USA; 2 Department of Neurosurgery, University of California, Los Angeles, USA; 3 Department of Psychiatry and Biobehavioral Sciences, University of California, Los Angeles, USA; 4 Department of Radiology, University of California, Los Angeles, USA

**Keywords:** Functional magnetic resonance imaging, Laser therapy, Diffusion tractography, Brain Neoplasms, Neuronavigation

## Abstract

LITT is a minimally-invasive laser ablation technique used to treat a wide variety of intracranial lesions. Difficulties performing intraoperative mapping have limited its adoption for lesions in/near eloquent regions. In this institutional case series, we demonstrate the utility of fMRI-adjunct planning for LITT near language or motor areas. Six out of 7 patients proceeded with LITT after fMRI-based tractography determined adequate safety margins for ablation. All underwent successful ablation without new or worsening postoperative symptoms requiring adjuvant corticosteroids, including those with preexisting deficits. fMRI is an easily accessible adjunct which may potentially reduce chances of complications in LITT near eloquent structures.

## Introduction

Laser interstitial thermal therapy (LITT) is a minimally invasive surgical ablation technique with shown efficacy in the treatment of primary and metastatic brain tumors, radiation necrosis (RN), and epileptogenic foci [16]. The advent of MR guidance [9] and real-time thermography [12] have made LITT an increasingly utilized tool among neurosurgeons. Initially used primarily for deep-seated lesions, LITT’s increasing indications include patient frailty, patient preference, and decreased morbidity in some cases relative to open surgery [13, 10, 17, 1]. Despite these advantages, LITT can still pose a risk of unintended thermal damage to adjacent normal brain tissue [14]. Near eloquent structures, LITT may cause postoperative deficits in motor or language function, even with the aid of MR guidance [18]. This is further exacerbated by the inability to perform cortical or subcortical mapping during ablation secondary to targeting/trajectory disturbances from physiologic movement. Therefore, many are investigating utilization of awake neurologic assessment [6] or non-invasive patient immobilization [11]. However, these additions are not without extra cost to the patient and increased discomfort during the prolonged procedure.

Given these limitations, our institution has adopted fMRI for preoperative language and motor mapping to better guide treatment choice and intra-procedural thermal safety. In this institutional case series, we present 7 patients with intracranial lesions in or near eloquent structures who underwent preoperative fMRI for consideration of LITT. By reporting clinical outcomes in this cohort, we aim to demonstrate an easily accessible adjunct measure for improving LITT outcomes in patients with high-risk lesions.

## Case series

**Fig. 1:**
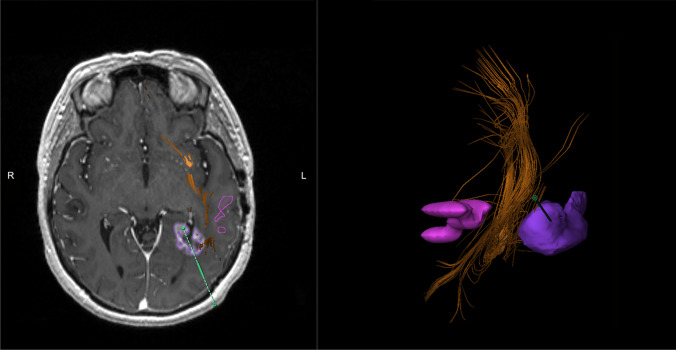
[Patient 4] fMRI demonstrating left temporal lesion (purple) without invasion of Wernicke’s area (pink) or the communicating superior portion of the temporal stem and orbital frontal lobe (orange); green dotted line = trajectory planning for laser ablation probe

**Fig. 2:**
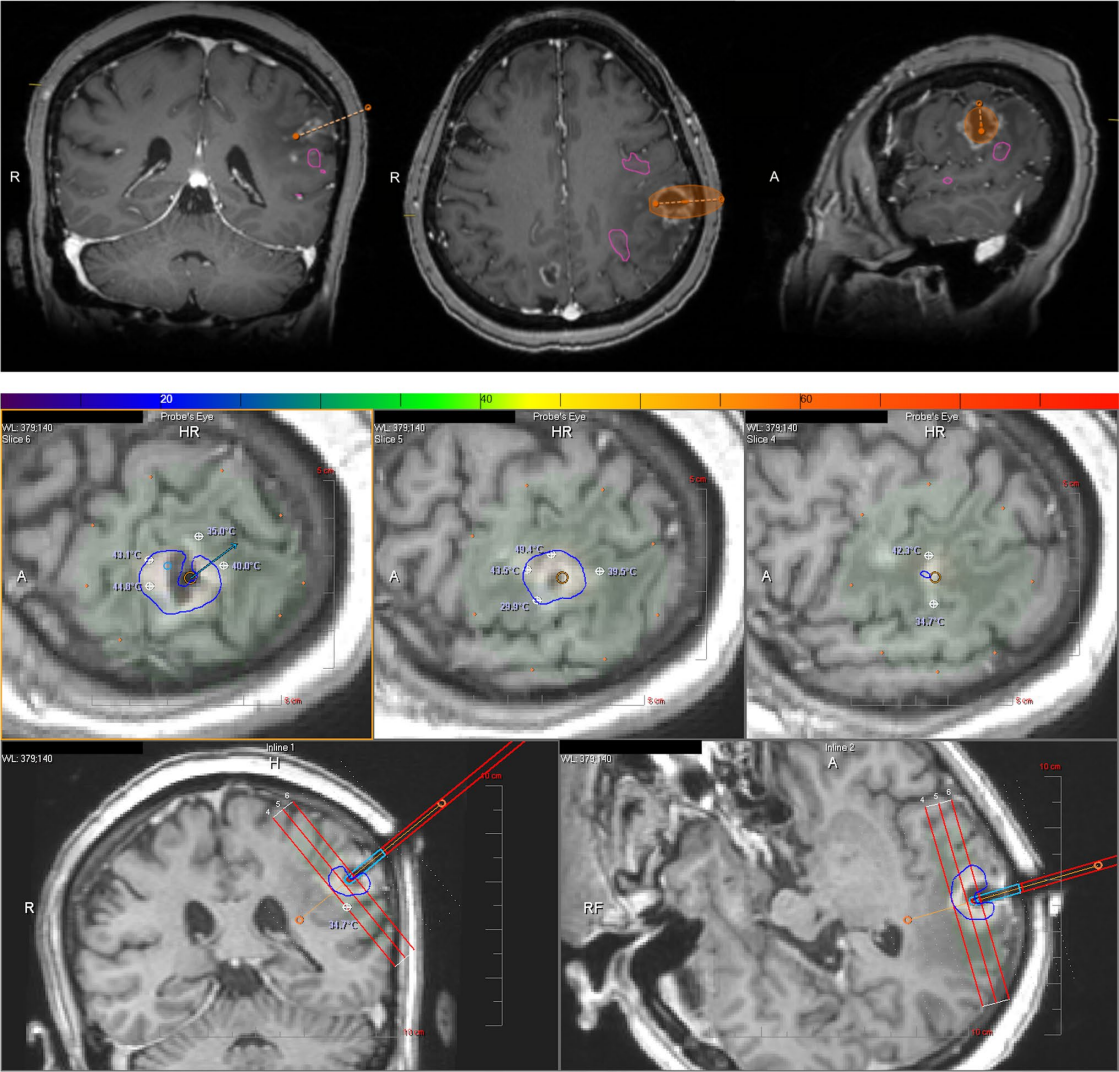
**a** [Patient 5] fMRI demonstrating left supramarginal gyrus lesion (orange) posterior to the postcentral gyrus without invasion of functional language areas (pink); orange dotted line = trajectory planning for laser ablation probe. **b** [Patient 5] Intraoperative temperature safety points (white crosshairs) and ablation safety margin (blue outline) using Monteris Neuroblate system

**Fig. 3:**
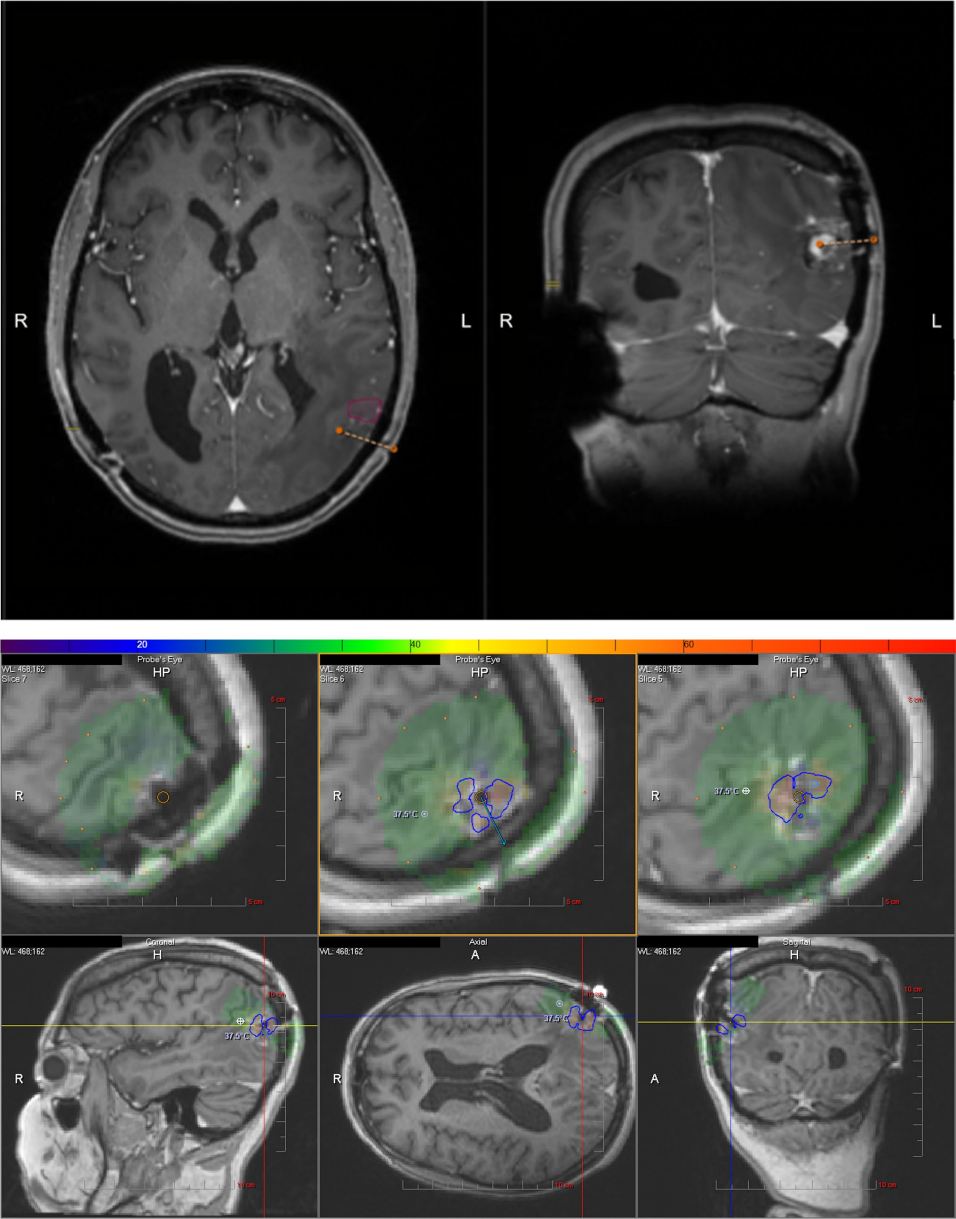
**a** [Patient 6] fMRI used to determine safety margin for a left parietal lesion near angular gyrus (purple) posterior to Wernicke’s area; orange dotted line = trajectory planning for laser ablation probe. **b** [Patient 6] Intraoperative temperature safety points (white crosshairs) and ablation safety margin (blue outline) using Monteris Neuroblate system

**Fig. 4:**
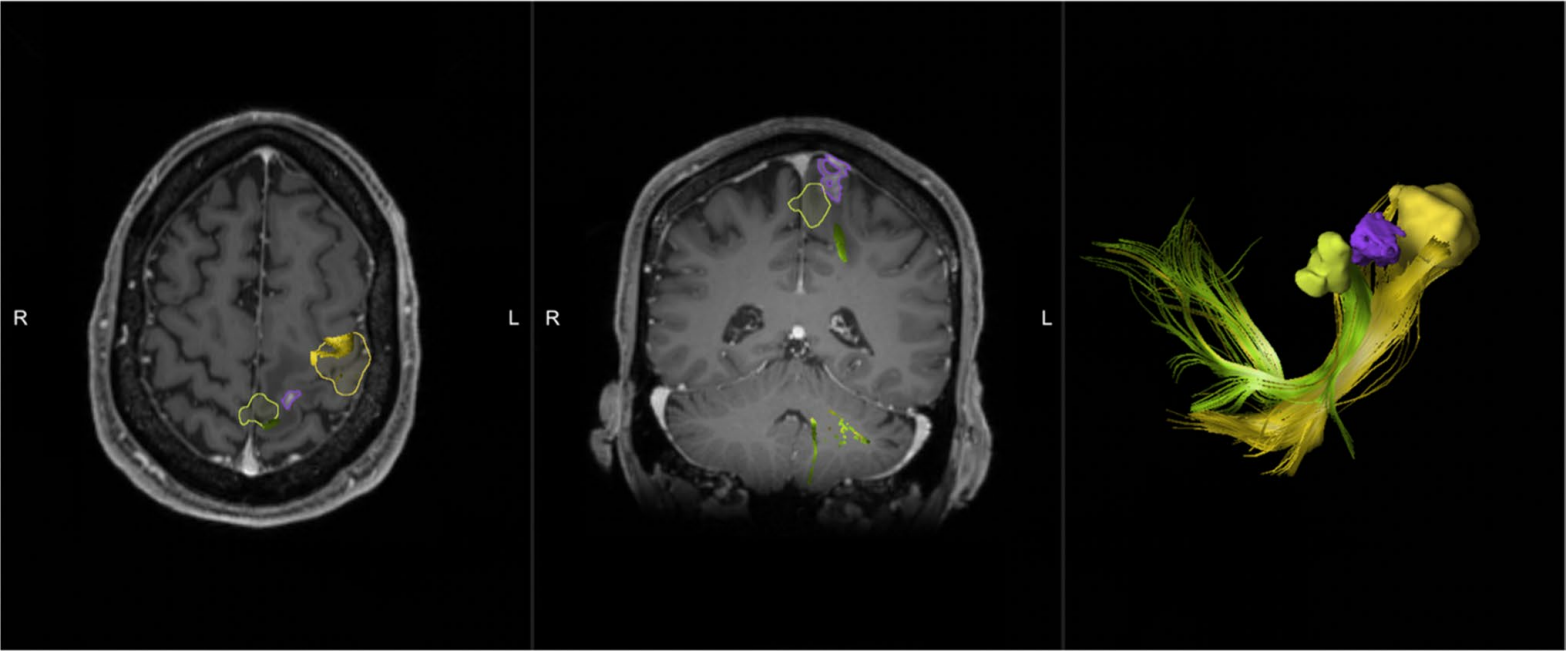
[Patient 7] fMRI demonstrating paracentral lesion too close to activation of leg motor (green) and hand motor (yellow) shown with respective subcortical tracts; patient underwent open resection with mapping instead of LITT

Patients who underwent LITT at our institution for any indication and fMRI prior to treatment (Neuroblate, Monteris Medical) were identified retrospectively with institutional IRB approval (Table 1). fMRI was performed to lateralize language, localize functional areas with respect to lesions, indicate risk of functional deficits, and determine feasibility and safety margins for LITT (Figs. 1, 2, and 3). Only one patient [Patient 7] did not undergo LITT due to significant eloquent involvement on fMRI analysis and ultimately succumbed to widespread disease progression (Fig. 4). Surgical outcomes and postoperative deficits were subsequently reported (Table 2).

**Table 1:** Summary of 7 fMRI-adjuvant LITT candidates including primary disease, lesion location, fMRI indication, and surgical decision making

Patient	Age	Sex	1º disease	Lesion location	fMRI used to assess proximity to:	Surgical decision
1	13	M	Ganglioglioma	L temporal	Wernicke’s area, arcuate fasciculus	Proceed with LITT
2	56	F	Breast cancer	L frontal	Primary motor cortex, corticospinal tract, Broca’s area	Proceed with LITT
3	17	F	Ganglioneurocytoma	R parietal	Primary motor cortex, corticospinal tract, language lateralization	Proceed with LITT
4	63	F	GBM	L peri-atrial	Wernicke’s area, arcuate fasciculus	Proceed with LITT
5	56	F	SCLC	R frontal L parietal	Left primary motor cortex, corticospinal tract, Wernicke’s area	Proceed with LITT
6	56	F	NSCLC	L temporo-parietal	Wernicke’s area, arcuate fasciculus	Proceed with LITT
7	51	M	NSCLC	L fronto-parietal	Left primary motor cortex, corticospinal tract, Wernicke’s area	Open resection with IOM

*GBM* glioblastoma multiforme, *SCLC* small cell lung cancer, *NSCLC* non-small cell lung cancer, *IOM* intraoperative monitoring

**Table 2:** Postoperative surgical and long-term clinical outcomes of fMRI-adjuvant LITT

Patient	Volume pre (cc)	Volume ablated (cc)	Residual (cc)	Pathology	Preop symptoms	Postop symptoms	Follow-up
1	0.466	0.466	0	*	Seizures, speech latency, verbal comprehension deficits	None	1 year
2	1.858	1.52	0.326	BM	None	None	1 year
3	†	†	†	*	Seizures	None	1 year
4	2.685	2.685	0	RN	Word-finding difficulties, expressive aphasia	None	2 months
5	2.143	1.978	0.165	RN	Word-finding difficulties, verbal/written comprehension deficits	None	1 month
6	1.27	0.927	0.379	RN	Tinnitus, gait instability, word-finding difficulties, short-term memory loss	None	5 months
7	2.32	2.32	0	BM	Seizures, right leg paresthesia	N/A**	4 months

*BM* brain metastasis, *RN* radiation necrosis, *N/A* not applicable. Dagger (†) means unable to assess due to imaging artifact from bolt. Asterisk (*) means biopsy not performed. Asterisks (**) mean patient deceased

## fMRI-guided tractography

Imaging was obtained on Siemens Prisma 3-Tesla MRI scanner. MRI protocol included high-resolution T1 and T2 images suitable for surgical navigation and a 64-direction DTI scan (*b* = 1000, 64 directions, 2 × 2 × 2 mm voxel dimension) for tractography. fMRI protocol included 96 T2* images (TE/TR/Voxel Size/FOV = 30/2.5/3.1 × 3.1 × 3/200 mm) with fat saturation. Matched-bandwidth high-resolution protocol was acquired at TE/TR/Voxel Size/FOV = 35/5.0/1.6 × 1.6 × 4/200 mm, 28 slices.

fMRI studies comprised three language tasks: an object naming and verb generation task, verbal responsive naming task, and auditory responsive naming task per previously described methods [2, 5]. Coplanar matched-bandwidth T2 images were collected before and after each scan for overlay in interpretation. fMRI preprocessing involved minimal data adjustments except for a 2-mm spatial smoothing kernel. Predicted BOLD correlation (canonical HRF and block design) was computed as Pearson’s *r*. Conjunctive AND combined threshold activation (*r* = 0.2, equivalent to *p* = 0.05) only on all 3 language scans in a set. The resultant activation map included only primary language areas with *p* < 0.000125. Functional maps were then overlaid as 3D objects onto coplanar matched-bandwidth MRI and DTI images using BrainLAB planning software. Deterministic tractography was done using the fMRI activation regions as fiber tract seeds. After the surgeon’s final review and trajectory addition, the completed plan was uploaded into BrainLAB Navigation hardware for surgery.

## Operative technique

Patients were placed in Mayfield head holders and underwent stereotactic registration (Brainlab). Stereotactic accuracy was improved using preoperative CT with Leksell head frame (*n* = 1), bone fiducials (*n* = 2), or intraoperative AIRO CT (*n* = 3). Within the BrainLab Elements station, a predetermined trajectory was planned along the lesion’s long axis. Multiple trajectories were planned if ablation width diameter exceeded 2 cm. A stereotactic-guided 4.5-mm burr-hole was created. Dura was punctured with a coagulative blunt probe, and stereotactic biopsy was performed. We secured Monteris’ LITT bolt into the burr hole then capped to maintain sterility during MRI transport. At MRI, the laser probe was sterilely driven through the bolt to target using Monteris robotic system. Probe position was confirmed on initial T1 with half-dose gadavist. Once confirmed, safety points were placed by the neuroradiologist (NS) onto the intraprocedural scan to represent the functional areas of activation from the fMRI, and ablation was performed with sequential probe movement until adequate ablation was achieved. A post-ablation T1 MPRAGE was obtained after administering the remaining contrast agent. Upon completion, the patient was returned to the MRI antechamber, the laser probe and bolt were removed, and the incision was closed.

## Discussion

In this series, we described our technique for using fMRI as an adjunct in cases with eloquent area lesions to help guide patient selection, operative planning, and intraoperative guidance for LITT. In our workflow, fMRI activation serves as the basis of segmentation of eloquent cortical areas. In contrast to standard, anatomical-based tractography, we use the fMRI validated cortical functional regions as the seeds for the subsequent segmentation of functional tracts. We then used these cortical and subcortical tract segmentations to guide the LITT trajectory and set heat threshold safety points during the ablation. With this approach, we saw good functional outcomes in our six LITT-treated patients without compromising EOA.

Sharma et al. [19] describe another approach to asleep LITT in eloquent areas. Their technique for LITT-ablation of a left thalamic GBM employed intraoperative transcranial motor evoked potentials (MEP), somatosensory evoked potentials (SSEP), and free-running electromyography (EMG) in a patient with preoperative right-sided weakness. They observed onset of right-sided EMG firing when the real-time temperature at the tumor border encroaching on the posterior limb of the internal capsule reached 40 °C, at which point they ended the ablation. Their patient had a stable exam apart from new right facial droop, which was not present immediately postoperatively, likely due to developing edema as opposed to direct thermal damage. Del Bene et al. describe their technique for IOM during LITT for lesions near the motor tract [4]. For preoperative planning, they integrate DTI and magneto-encephalography to serve as a functional tractographic roadmap, similar to how we use fMRI and DTI tractography in our practice. Intraoperatively, in addition to SSEP and MEP monitoring, they performed microelectrode subcortical monopolar stimulation along the planned LITT probe tract to map proximity to motor tracts. From this, they extrapolated the ablation safety radius using the 1 mA = 1-mm framework. They describe the use of this approach to treat a previously radiated, asymptomatic rolandic BM without any new postoperative deficits.

Two groups have described their experience with awake LITT. In a 10-patient series, Laurent et al. used thermoplastic masks to facilitate head stabilization during ablation [11]. They do not comment on proximity to eloquent regions. One patient had an intra-procedural seizure which abated with anti-epileptics, and four patients required rescans following patient movement. Hajtovic et al. report a series of 6 patients with eloquent tumors undergoing intraprocedural monitoring of speech, motor, or visual function every 5-min during ablation [6]. In this awake series, 5/6 patients were immobilized in stereotaxic frames. They report no intraoperative seizures or instances of rescanning due to patient movement. Based on these early experiences, awake LITT may be feasible in experienced centers, but not without challenges unique to LITT (e.g., importance of patient immobility) and common to awake neurosurgery more generally (e.g., risk of intraoperative seizures, patient discomfort).

Regardless of approach used for LITT in awake patients, even with accurate targeting and avoidance of direct thermal damage to eloquent structures, some patients do experience delayed neurological dysfunction. Transient cytotoxic edema following ablation is believed to cause temporary perioperative deficits or worsen preexisting deficits [14, 18, 20], which often necessitates postoperative steroids to resolve [3, 8, 7]. Within our cohort, 4/6 patients were on steroids at time of procedure. Steroid treatment was continued post-procedure and tapered gradually. The other two patients were placed on short dexamethasone tapers (< 1 week) post-procedure. None of the patients had to increase dosage or restart steroids after end of taper due to new symptoms. This is an unusual finding, as virtually all the aforementioned studies reported cases of new or worsened perioperative deficits which responded to corticosteroids. Even with the use of awake testing, Hajtovic et al. reported 3/6 patients experienced delayed neurological deterioration 2–3 weeks post-ablation, which resolved with subsequent steroid treatment. Patients should be advised on the possibility of delayed deficits, and that these often respond to corticosteroids and abate with time. These may be more common in those with preoperative deficits [14, 8, 15].

While our cohort is small, the utility of adjunct fMRI for pre- and intra-operative planning is promising. Tractography based on fMRI activation can guide safe ablation near eloquent regions without awake testing. Our cohort showed consistent perioperative improvement, with no evidence of permanent neurological deficits. Further studies with larger cohorts and more extensive follow-up periods are required to better understand the long-term outcomes of fMRI as adjunct planning for LITT near eloquent structures.

## Data Availability

Not applicable.
